# Protein plasticity driven by disorder and collapse governs the heterogeneous binding of CytR to DNA

**DOI:** 10.1093/nar/gky176

**Published:** 2018-03-10

**Authors:** Sneha Munshi, Soundhararajan Gopi, Sandhyaa Subramanian, Luis A Campos, Athi N Naganathan

**Affiliations:** 1 Department of Biotechnology, Bhupat & Jyoti Mehta School of Biosciences, Indian Institute of Technology Madras, Chennai 600036, India; 2 National Biotechnology Center, Consejo Superior de Investigaciones Científicas, Darwin 3, Campus de Cantoblanco, 28049 Madrid, Spain

## Abstract

The amplitude of thermodynamic fluctuations in biological macromolecules determines their conformational behavior, dimensions, nature of phase transitions and effectively their specificity and affinity, thus contributing to fine-tuned molecular recognition. Unique among large-scale conformational changes in proteins are temperature-induced collapse transitions in intrinsically disordered proteins (IDPs). Here, we show that CytR DNA-binding domain, an IDP that folds on binding DNA, undergoes a coil-to-globule transition with temperature in the absence of DNA while exhibiting energetically decoupled local and global structural rearrangements, and maximal thermodynamic fluctuations at the optimal bacterial growth temperature. The collapse is shown to be a continuous transition through a combination of statistical-mechanical modeling and all-atom implicit solvent simulations. Surprisingly, CytR binds single-site cognate DNA with negative cooperativity, described by Hill coefficients less than one, resulting in a graded binding response. We show that heterogeneity arising from varying binding-competent CytR conformations or orientations at the single-molecular level contributes to negative binding cooperativity at the level of bulk measurements due to the conflicting requirements of collapse transition, large fluctuations and folding-upon-binding. Our work reports strong evidence for functionally driven thermodynamic fluctuations in determining the extent of collapse and disorder with implications in protein search efficiency of target DNA sites and regulation.

## INTRODUCTION

Thermodynamic fluctuations of macromolecules are one of the primary factors driving transcription, binding reactions, allostery and thus the cellular responses to varied environmental conditions (1–7). Macromolecules are therefore highly sensitive to temperature modulations with the extent of protein oligomerization, degradation and protein disorder all dependent on temperature (8,9) and slaved to solvent composition, structure—extent and strength of hydrogen bonds in the bulk versus the first shell—and motions (10). It is also now well established that thermodynamic stability is a continuum ranging from intrinsically disordered (IDPs) to well-folded rigid proteins; experimentally, the measured stability (with reference to the folded state) can be negative (i.e. only unfolded like conformations are populated) to 10–15 *RT*. The large conformational heterogeneity of IDPs contributes to moonlighting functions (11–13), facile regulation (14,15), ‘fly-casting’ effects (16), structural changes driven by post-translational modifications (17), phase separation (18), fuzzy complexes (19), domino-like destabilization (20) and many more (21,22).

In systems that are reasonably well-folded, changes in thermal fluctuations at lower temperatures (<320 K) can modulate the population, reconfiguration dynamics within and kinetics between partially structured, intermediate and even the unfolded states. On the other hand, unfolded or disordered proteins collapse with temperature (23–25) irrespective of their sequence composition that can range from glycine-serine or poly-glutamine repeats (26), glycine-rich peptides (27) to proteins rich in hydrophilic residues (28). One of the many questions that has kindled the interest on disordered proteins is the effect of changing solvent conditions on the dimensions of disordered polypeptide chains to test for various polymer scaling laws that relate radius of gyration (*R_G_*) to protein length (*N*), i.e. ${R_G} = {\rho _0}{N^\nu }$ where *ν* is the Flory exponent (29). The exponent ranges from 1/3 for compact conformations (poor solvent), 1/2 for Θ-point conditions (ideal- or random walk chain) to 3/5 for expanded coils (or self-avoiding random walk chains in good solvent) (30,31). Detailed experiments on multiple disordered proteins suggest that under native-like conditions protein chains are close to the Θ-state where chain–chain and chain–solvent interactions are effectively balanced (32).

The discussion above raises interesting questions. Are disordered or unfolded protein collapse transitions first- or second-order-like? Since disordered systems exhibit no tertiary packing interactions and little secondary structure, if any, it has been proposed that IDPs undergo second-order-like transitions or continuous or downhill unfolding (33,34), i.e. the free energy landscape of such systems are titled towards unfolded like conformations without any macroscopic free energy barrier separating the varied conformations. If disordered polymeric chains indeed undergo a second-order like collapse transition as expected from the unimodal FRET efficiency histograms, they should exhibit thermodynamically decoupled structural transitions, as shown for folded proteins with weak energetic coupling (35–37), that has not been explored before for any IDP. At critical or collapse transition points, the free energy surfaces are flat in continuous transitions contributing to large thermodynamic fluctuations. Accordingly, there should be a near one-to-one correspondence between Θ-state and thermodynamic fluctuations.

**Figure 1.**
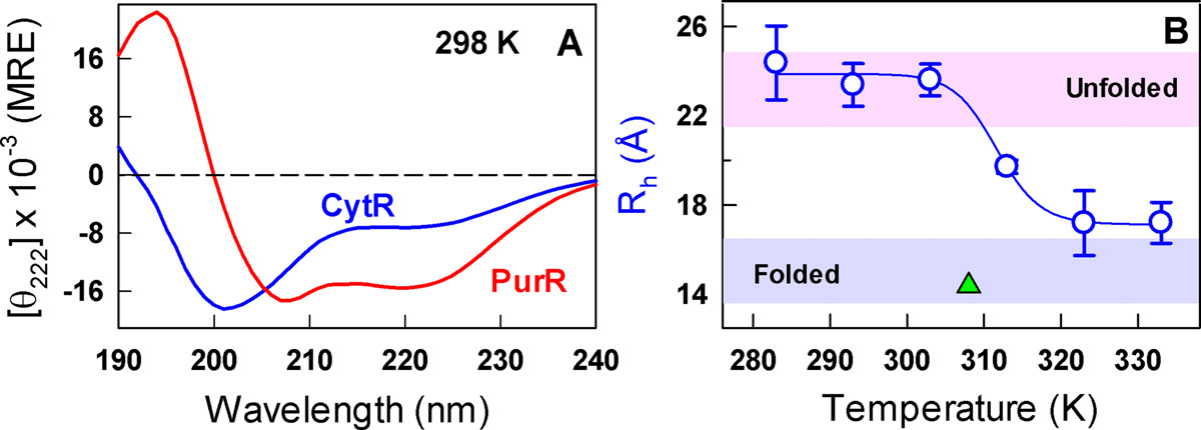
Disorder and collapse. (**A**) Far-UV CD spectra of CytR (blue) and PurR (red) at 298 K in mean residue ellipticity units of deg. cm^2^ dmol^−1^. (**B**) Changes in the hydrodynamic radius (*R_h_*) of CytR with temperature extracted from dynamic light scattering measurements. The curves are shown to guide the eye. Shaded regions in pink and blue represent the hydrodynamic radius of disordered and ordered proteins, respectively, from size-scaling expectations (44). The green triangle represents the *R_h_* of CytR from the DNA-bound structure at 308 K calculated using HYDROPRO (45).

The near-universal observation of collapse in disordered systems further raises the question of whether nature fine-tuned the extent of conformational heterogeneity in disordered proteins for modulating the binding affinity to their partner ligands. If so, the large fluctuations implicit under such conditions might result in non-trivial functional output with potential promiscuous binding, a feature that is increasingly being observed in disordered systems. We explore these issues in the current work by studying the monomeric and helical DNA-binding domain of the prokaryotic protein CytR (cytidine repressor) that regulates the expression of proteins responsible for nucleoside recycling (38,39). This is a unique system in that CytR is disordered (Figure 1A) despite exhibiting high sequence complexity and folds upon binding to its target sequence (*udp* promoter), highlighting an extreme case of disorder-to-order transition (40).

Through a combination of experimental multi-probe spectroscopic studies, accurate heat capacity measurements, a statistical model and all-atom implicit solvent simulations we show strong evidence that CytR follows a second-order-like but non-specific collapse transition with temperature. We then test whether chain collapse is a functionally driven feature encoded in disordered proteins by probing the binding ability of different conformational states of CytR to its partner DNA. We identify that at the optimal bacterial growth temperature, CytR has multiple conformational states to choose from - expanded, collapsed, folded-like. This frustration results in an array of binding competent poses, some specific and some non-specific, thus translating into a graded response of the binding signal.

## MATERIALS AND METHODS

### Equilibrium spectroscopy

All experiments on CytR and PurR were recorded in freshly prepared, filtered and degassed 20 mM sodium phosphate buffer at pH 7.0 without or with urea, unless mentioned otherwise. Protein solutions were routinely filtered through 0.22 μm syringe filters before every experiment. Far-UV and near-UV circular dichroism (CD) spectra were recorded in a Jasco J-815 spectropolarimeter connected to a Peltier system at a concentration of ∼25 and ∼100 μM, respectively. The urea melts in the range of 0 to 6–8 M urea were recorded at 298 K under the same buffer conditions as above. The fluorescence experiments were performed in a Chirascan Plus qCD instrument (Applied Photophysics Ltd., UK) by exciting ∼26 μM of CytR in 10 × 10 mm pathlength cuvette at 274 nm and collecting the spectra between 280 and 400 nm. The quantum yields were estimated employing NATA as a reference (0.13 at 298 K in water).

### Dynamic light scattering (DLS)

The changes in dimensions of CytR with temperature were monitored using a DynaPro-MS/X instrument (Protein Solutions, Charlottesville, VA, USA) coupled to a Peltier temperature controller. CytR samples at different concentrations (0.45, 0.90 and 1.35 mg/ml) were first centrifuged at 10000g for 10 minutes and then filtered with a 0.1 μm filter (Anotop 10, 0.1 μm from Whatman). 50 μl samples were loaded on to a cuvette and the scattered light intensity was collected at 90° at different temperatures (from 283 to 333 K, every 10 K). The translational diffusion coefficients (*D*) were determined from the time-series scattering data using the DYNAMICS autocorrelation analysis software v.6 (Protein Solutions). To account for solution non-ideal effects, the diffusion coefficients were measured at different protein concentrations (*C*) and the resulting *D* values were fit to an equation of the form $D = {D_0} \cdot (1 + a \cdot C)$ where *D*_0_ is the intercept. The *D*_0_ and the *D* measured at the lowest protein concentration were employed to estimate the hydrodynamic radius (*R_h_*) from the Stokes–Einstein relation and the temperature-dependence of water viscosity.

### Differential scanning calorimetry (DSC)

Heat capacity thermograms were recorded in a MicroCal VP-Capillary DSC with an automated sample injector. The instrument was thoroughly equilibrated with multiple buffer scans following which CytR protein solutions ranging from ∼110 to 52 μM were scanned thrice at a scan rate of 1 K/min. Buffer-buffer baselines were routinely recorded before and after every protein scan to check for instrumental baseline drifts. The absolute heat capacity was then determined as described by Sanchez-Ruiz and coworkers (41).

### Variable barrier (VB) model

The VB model analysis was performed employing the absolute heat capacity thermogram of CytR as described in the original work (42). The final parameters are: Σ*α* = 1554.9 kJ mol^−1^; characteristic temperature, *T*_0_ = 291.7 K; *β* = −173.7 kJ mol^−1^ (together with Σ*α* it determines the shape of the free energy profile at *T*_0_ in downhill profiles; it represents the barrier height in a two-state-like system); asymmetry factor, *f* = 0.535.

### Protein–DNA binding experiments

Double stranded udpO (5′-ATT**TATGCAAC**GCA-3′) tagged with ALEXA532 at 5′ end was purchased from IBA Lifesciences (the binding site is highlighted in bold). Experiments were performed in 50 mM sodium phosphate, 30 mM sodium chloride and 1 mM EDTA, pH 6.0 buffer. A starting DNA concentration of 300 nM was titrated with different concentrations of CytR ranging from ∼1 nm to 100 μM; the resulting change in anisotropies were monitored following a 5 minute equilibration at every titration step by exciting the dye at 530 nm and collecting the emission at 580 nm in a Chirascan Plus qCD instrument (Applied Photophysics Ltd., UK) equipped with a fluorescence polarization accessory.

### Implicit solvent replica exchange MC (REMC) simulations

Simulations were performed using CAMPARI stand-alone package, employing ABSINTH implicit solvent model with OPLS charges (43). The protein was placed in a 100 Å spherical shell with 105 explicit excess ion-pairs to simulate 43 mM ionic strength conditions. REMC simulations were carried out with 20 temperature replicas, ranging from 280 to 430 K, with exchange attempts every 20 000 steps resulting in an average exchange probability of ∼0.4 across temperatures. Simulations were carried out starting from the randomized initial structure with temperature-dependent dielectric constant and solvation free energies as before (28). Each of the replicas was run for 6 × 10^7^ steps in parallel; snapshots were collected every 500 steps and the first 3 × 10^7^ steps were discarded from further analysis. One-dimensional free energy profiles and two-dimensional surfaces were generated using the weighted histogram analysis method (WHAM).

## RESULTS

### Dimensions of coil and collapsed globular states

To quantify the extent to which the overall dimensions of CytR change with temperature, we measured the hydrodynamic radius through non-invasive dynamic light scattering (DLS) experiments that do not require fluorescent probes. The DLS experiments were performed at different concentrations of the protein at each temperature from which the effective hydrodynamic radii were extracted (see Materials and Methods). As expected from the crowded NMR spectra (40), the *R_h_* of CytR is found to be ∼24 Å at the lowest temperature with the dimensions matching the size-scaling empirical relationship of Uversky *et al.* (*U_E_*; shaded regions in Figure 1B) (44). Upon temperature increase the overall *R_h_* decreases reaching a value of ∼17 Å at 333 K with a collapse temperature (*T*_c_) of ∼312 K. Interestingly, the collapsed globule-like state (*U_C_*) of CytR exhibits a hydrodynamic radius similar to that of the folded protein in the presence of its cognate DNA (∼15 Å; green triangle in Figure 1B). The small difference in *R_h_* (∼12%) between the fully collapsed state and the folded state is in agreement with the relative collapsed state dimensions observed upon dilution of denaturants in single-molecule FRET measurements on folded proteins (30).

### Thermodynamically decoupled conformational changes

**Figure 2.**
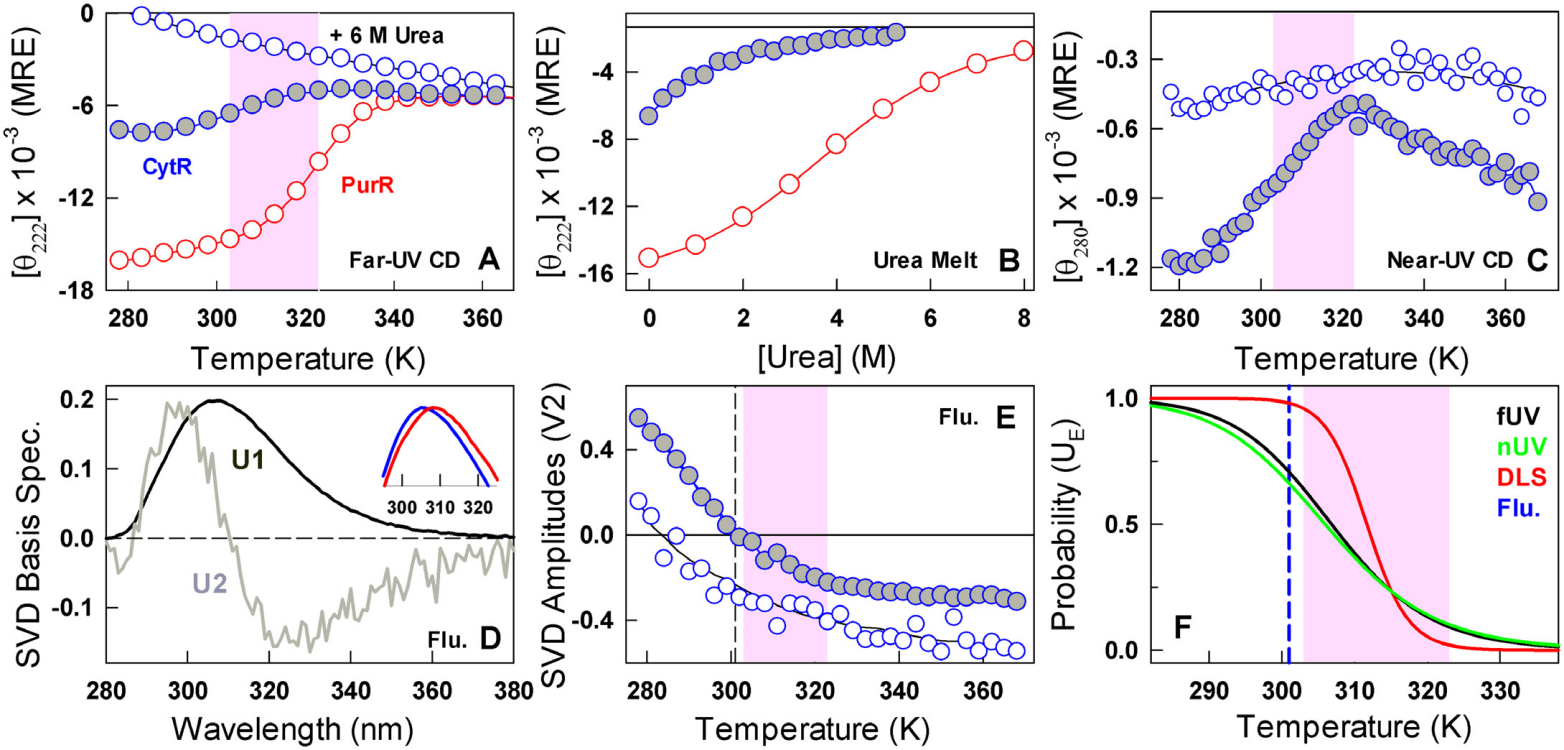
Multi-probe characterization of CytR conformational changes. Filled and open blue circles are CytR in 43 mM ionic strength conditions and 6 M urea (both at pH 7.0), respectively. Red circle represents PurR at pH 7.0, 43 mM ionic strength conditions. Shaded areas highlight the collapse transition regime in CytR. (**A**) Temperature dependent changes in secondary-structure reported in mean residue ellipticity (MRE) units of deg. cm^2^ dmol^−1^. (**B**) Urea melts at 298 K highlighting the differences in the unfolding patterns of disordered CytR and folded PurR, respectively. (**C**) Changes in the tertiary structural environment monitored by near-UV CD of Y53 at 280 nm. (**D**) Basis spectra from a global singular value decomposition (SVD) of temperature-wavelength fluorescence emission of CytR in both 43 mM buffer and 6 M urea conditions. The first component reports on the average spectrum (U1) while the second component (U2) represents the red shift of the emission profile. Inset: normalized emission spectra of CytR at 278 K (blue) and 350 K (red) at 43 mM buffer, pH 7.0. (**E**) Amplitudes (V2) of the second basis spectrum shown in panel D. The vertical dashed line reports on a spectral sign change beyond which emission broadening dominates the observed spectrum. (**F**) Apparent probabilities of expanded coil conformations (*U_E_*) as a function of temperature from a two-state analysis of the observed far-UV CD (black), near-UV CD (green) and DLS (red) transitions. The inflection point for the tyrosine red shift is shown in blue.

Most disordered proteins exhibit little or no secondary structure. Moreover, as the temperature is increased the mean residue ellipticity at 222 nm generally becomes more negative suggestive of higher secondary structure content or more likely due to a decreased propensity for polyproline like conformations (46). The disordered CytR, on the other hand, displays secondary structure loss with temperature (Figure 2A) in sharp contrast to the signal at 6 M urea. As a reference, we studied the homologous DNA-binding domain from PurR that exhibits a sequence similarity of 64% with CytR (identity of 48%); we find that PurR is well folded (Figure 1A) and exhibits a sigmoidal-like unfolding curve at the same experimental condition (Figure 2A). The respective chemical denaturation induced unfolding profiles at 298 K indicate a loss of secondary structure with different apparent cooperativities expected of a disordered and folded protein, respectively (Figure 2B). The comparison highlights that disordered CytR populates helical-like secondary structures at low temperature that are lost at higher temperatures. It is also clear that the observed differences originate from specific differences in the primary sequence between the two proteins and not from the choice of experimental conditions.

The sole tyrosine in CytR (Y53) provides another probe to monitor the collapse transition. CytR at 6 M urea reveals little changes in the near-UV CD signal at 280 nm. CytR in the absence of urea, however, shows a weak near-UV CD spectrum that further loses intensity with increasing temperature mimicking far-UV CD observations (Figure 2C). In both cases, the apparent midpoint of the collapse transition appears a bit earlier thermodynamically (compared to DLS) at ∼307 K when employing a two-state-like equilibrium model between *U*_E_ and*U_C_*. We also observe a red shift in the fluorescence emission of tyrosine with temperature, a phenomenon that is rarely reported in proteins; this could be indicative of a temperature-driven transition between sub-ensembles that populate folded-like to more unfolded-like conformations. This in turn would have an effect on the fluorescence emission of Y53 because of changes in either its immediate tertiary environment (Figure 2C) or through specific differences in nature and strength of hydrogen bonding involving the tyrosyl group. Evidence for this is observable in the fact that the red shifted emission is dominant at even lower temperatures for CytR at 6 M urea (i.e. when the ensemble is highly unfolded) while it dominates the emission only at a higher temperature for CytR at pH 7.0, 43 mM ionic strength conditions (Figure 2D and Supplementary Figure S1). This structural change also happens thermodynamically much earlier than either DLS or CD with an apparent midpoint of ∼301 K (Figure 2E).

The observations above can be summarized in a representation that highlights the variable transition midpoints from different experimental probes in the range of experimental temperatures (Figure 2F). Interestingly, significant structural changes precede the actual collapse transition than within the narrow range of ∼20 K monitored by DLS.

### Thermodynamic fluctuations peak at the collapse transition midpoint

The large differences in the apparent collapse transition temperatures are indicative of an energetically decoupled system where different parts of the structure undergo structural changes independent of one another upon temperature modulations, reminiscent of a second-order-like collapse transitions in homo-polymeric systems or proteins in the globally downhill folding regime (35,36). Scanning calorimetry experiments (DSC) provide a powerful avenue to explore the nature of the collapse transition as they are probe-independent and report on the overall thermodynamics (47). Moreover, it is possible to extract the precise nature of the collapse transition—specific or non-specific and first- or second-order?—given the intrinsic connection between the order of transition and the enthalpic fluctuations reported by DSC (42). However, these experiments are challenging to perform given the small excess heat associated with structural transitions in disordered systems and the necessity to generate absolute heat capacities that requires multiple experiments at different protein concentrations.

**Figure 3.**
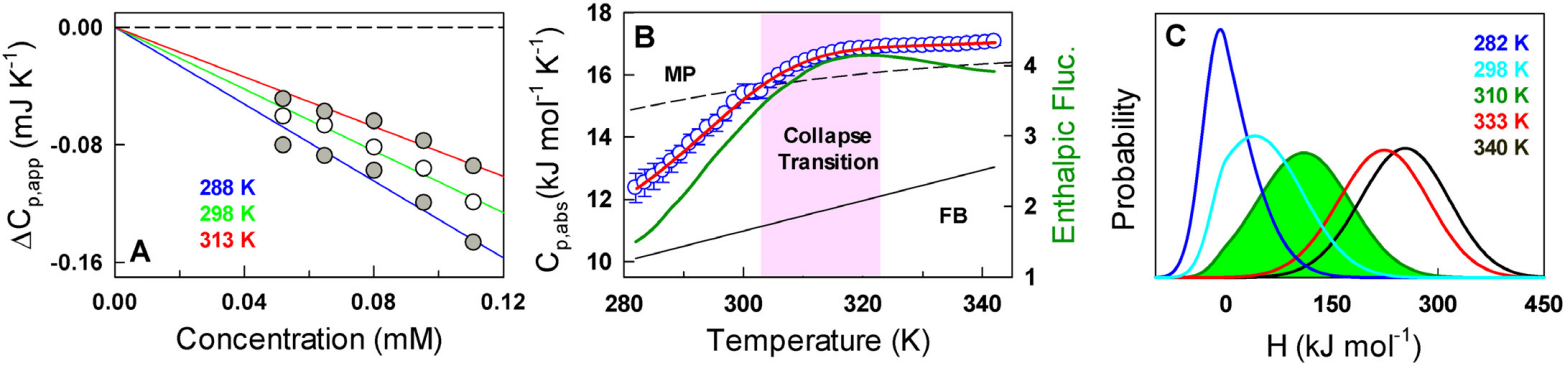
Thermodynamic fluctuations and the nature of the collapse transition. (**A**) Apparent heat capacities as a function of concentration (circles) together with the linear fit (lines) from which absolute heat capacities are extracted. (**B**) The absolute heat capacity (blue circles) together with the folded Freire (continuous black) and unfolded Makhatadze-Privalov (MP; dashed black) baselines. Red curve is a fit to the experimental data derived from the variable barrier model. The dark green curve (right ordinate) represents the temperature dependent excess enthalpic fluctuations of the system in units of (kJ mol^−1^)^2^. (**C**) Probability distribution of microstates as a function of enthalpy as the order parameter. Note that the densities are unimodal at all temperatures.

CytR is highly soluble (up till 300 μM) and the thermograms highly reversible enabling us to obtain precise concentration-dependent apparent Δ*C_p_* (Figure 3A) from which absolute heat capacities were extracted following the protocol prescribed by Sanchez-Ruiz and coworkers (41). The absolute heat capacity profile of CytR reveals no pre-transition region, no sharp excess heat capacity associated with barrier-limited transition, and is well bounded by the empirical folded Freire and unfolded Makhatadze-Privalov (MP) baselines (Figure 3B) (48,49). This is, to the best of our knowledge, the first absolute heat capacity measurement on a disordered protein system. The unique features are characteristic of a continuous second-order-like transition from an unfolded but extended collection of conformations to a collapsed state. Enthalpic fluctuations derived from the excess heat capacities (i.e. after subtracting the native baseline that includes contributions from non-conformational fluctuations of the system) indicate that the collapsed globule (*U_C_*) undergoes large amplitude enthalpic fluctuations (even more than the *U_E_*) arguing against a specifically collapsed state (dark green in Figure 3B). Moreover, the fluctuations are maximal at the midpoint of the collapse transition (∼312 K) indicating a system that is potentially frustrated between multiple conflicting interactions. An analysis of the thermogram employing the phenomenological variable-barrier model (42), which is based on the Landau theory of phase transitions, provides a quantitative picture of the underlying thermodynamic landscape (see Materials and Methods). The probability density continuously shifts from low enthalpy to high enthalpy states with no evidence for two macrostates coexisting at any condition thus ruling out a barrier-limited two-state transition (Figure 3C).

DSC experiments therefore provide strong evidence for a continuous or downhill-like collapse transition in CytR. The maximal enthalpic fluctuations and hence the maximal structural heterogeneity are observed at temperatures ∼310–313 K. Since the polymeric chain is expected to exhibit no preferential intra-chain or chain-solvent interactions under these conditions (Θ-state) (29), the dimensions of CytR should match the expectation of an ideal chain. To check for this, we convert the measured hydrodynamic radius values to radius of gyration following a recent procedure (50) resulting in a change in *R_G_* from ∼26 Å in good solvent conditions (low temperature) to ∼12 Å in poor solvent (high temperature) (Supplementary Figure S2). At the critical point of 312 K, the extracted *R_G_* value is ∼17 Å, close to the expectation for a polymer in its Θ-state (∼17.9 Å) from ${R_G} = 2.2{N^{1/2}}$ (in Å units and for *N* = 66) (32).

### A flat landscape with multiple conformational sub-states from all-atom simulations

**Figure 4.**
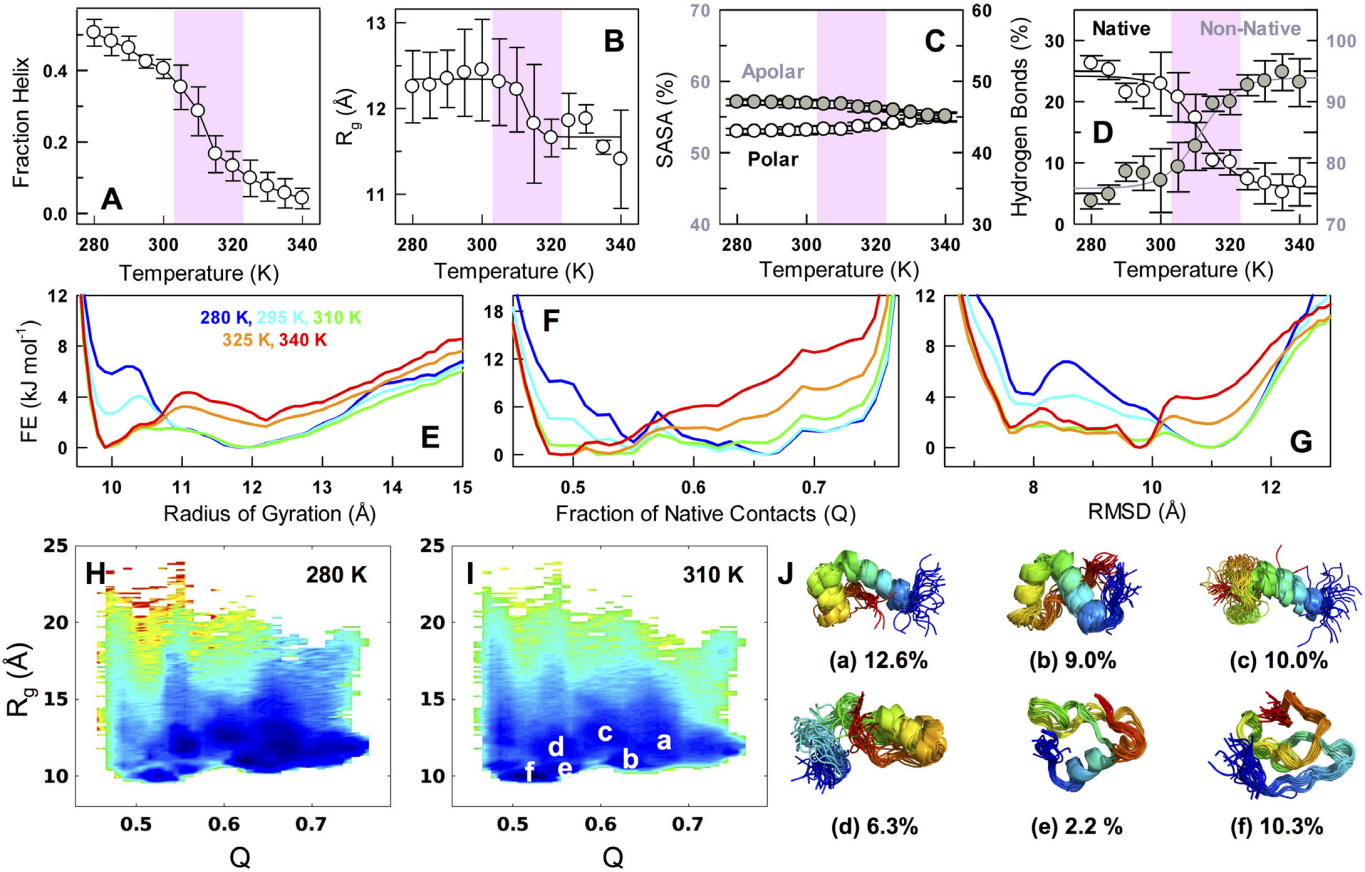
Continuous collapse and the nature of ensembles from all-atom simulations. (**A**–**D**) Fraction helix, radius of gyration (*R_g_*), solvent accessible surface area (SASA) and the fraction of hydrogen bonds with temperature. The equilibrated ensemble of structures is split it into two equal parts and the observable is calculated within each to generate error bars. The shaded area represents the collapse transition regime as identified in experiments. (**E**–**G**) One-dimensional free energy profiles as a function of *R_g_*, fraction of native contacts (*Q*; with respect to the folded structure) and root mean square deviation (backbone RMSD with respect to the folded structure). Small barriers of the order of 1–3 kJ mol^−1^ arise due to the reduction in dimensionality. The wells are maximally broad, i.e. largest heterogeneity, at 310 K (green). (**H**–**I**) Projection of the conformational ensemble onto two order-parameters—*R_g_* and *Q*—and color-coded in the spectral scale (blue to red represents low to high free energy). (**J**) A structural view of the most populated sub-ensembles at 310 K (superimposition of 28–31 frames in each) and identified in white in panel I. The sub-ensemble *f* is a collapsed state with no secondary structure.

To explore the nature of transition further and to identify the conformational characteristics of the ensembles that are populated, we performed all-atom implicit solvent replica-exchange Monte Carlo (REMC) simulations employing the ABSINTH implicit solvent model (43) that includes specific temperature-dependent solvation parameters (28). Remarkably, the collapse transition midpoint and the helical nature of the low-temperature ensemble are semi-quantitatively reproduced by the simulation methodology without any modification to the default simulation parameters (Figure 4A and B). Control simulations that do not incorporate temperature-dependent solvation free energies fail to capture the collapse transition in agreement with the results of Schuler *et al.* (Supplementary Figure S3) (28). Very little change is observed in the polar-apolar solvent accessible surface area of the disordered ensemble on temperature changes (Figure 4C). However, there are two significant differences when compared to experiments: the degree of predicted helical structure is much higher at low temperatures and the amplitude of the collapse transition is modest compared to that observed in experiments (30% change). Given the agreement at the *de-novo* level, we construct multiple 1D and 2D projections to extract the order of the collapse transition. The underlying landscape is expectedly rough but with minimal or zero thermodynamic barrier separating the numerous states in all of the projections including radius of gyration, number of native contacts (Q; with the folded structure as a reference) and backbone RMSD (Figure 4E–I, Supplementary Figure S4). This observation is in line with the expectation of a second-order-like transition from current experiments, theoretical predictions on CytR (51) and the unimodal probability densities of end-to-end distance distributions from numerous disordered proteins. At 310 K, a range of structures are populated that include partial helical structure in all native helices or a few of them (Figure 4J) similar to that observed from a structure-based statistical mechanical model (51). In addition to these, a collapsed sub-ensemble with little secondary-structure is also evident (*f* in Figure 4I, J) highlighting the complex phase space accessible under these conditions; the population of this sub-ensemble increases with temperature contributing to the collapsed and compact globule (panel C in Supplementary Figure S5).

How is the collapsed state stabilized? We find here that the fraction of native hydrogen bonds (those present in the folded CytR structure) decrease with temperature while the fraction of non-native hydrogen bonds increase with temperature (Figure 4D). The latter primarily involves the protein backbone and side-chain interactions involving the uncharged polar amino acids of Asn, Gln, Ser and Thr. It is important to note that these simulations have been performed with a temperature-dependent solvation free energy that has been shown to capture the collapse transition in multiple proteins. Therefore, while solvation could be a major factor determining the collapse, the resulting collapsed state seems to be energetically stabilized by multiple non-native hydrogen bonds. Given that CytR loses helical structure with temperature, these observations immediately highlight that the balance between the energetic terms determining helical structure and the solvation terms need more precise calibration for better agreement with experiments.

### Binding heterogeneity

**Figure 5.**
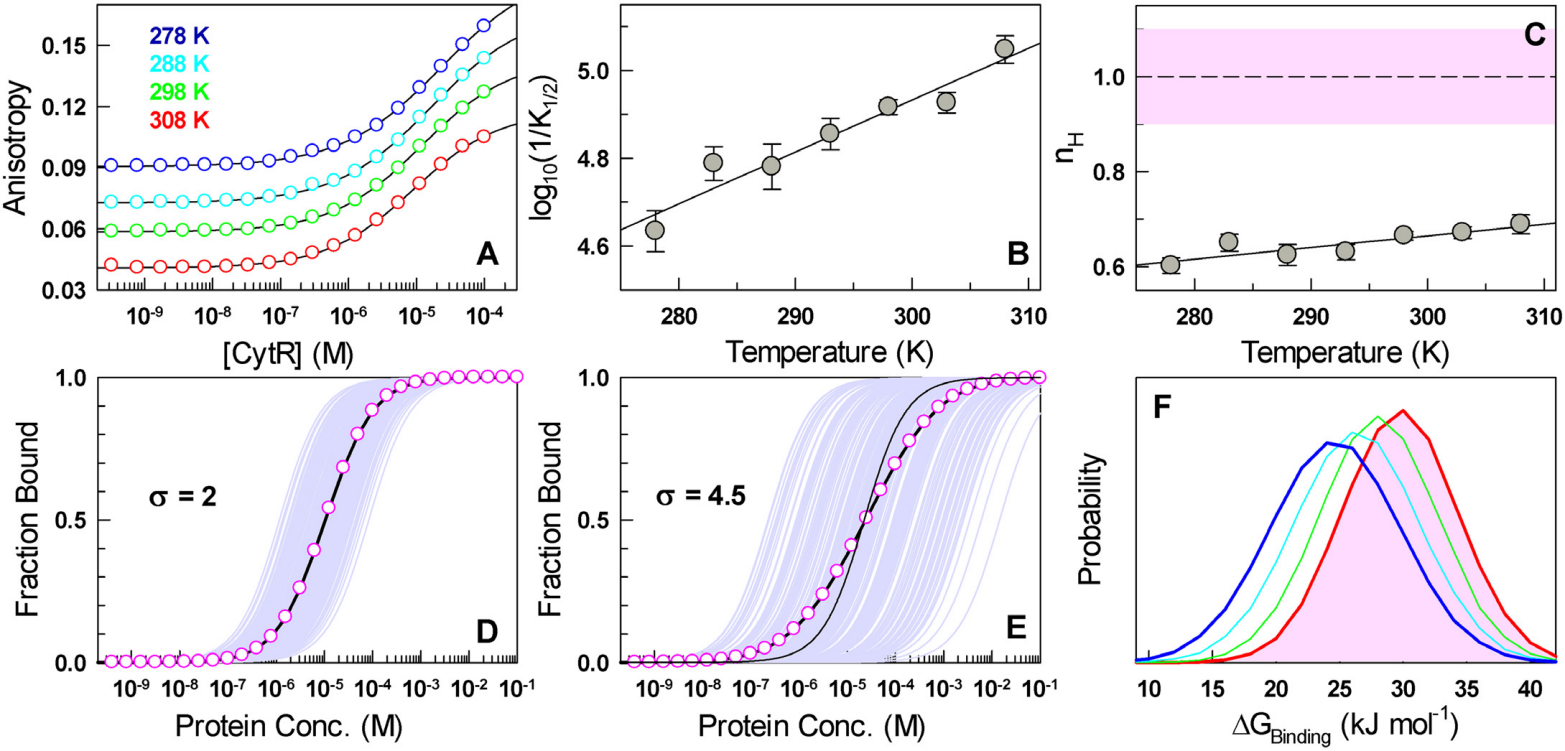
Binding heterogeneity explains negative cooperativity in 1:1 binding. (**A**) Anisotropy of dye-conjugated DNA as a function of CytR concentration at different temperatures (circles). Black curves are from fits to the Hill equation. (**B**) The apparent binding affinity (1/*K_1/2_*) as a function of temperature. The line is shown to guide the eye. (**C**) The Hill coefficients (*n_H_*) as a function of temperature together with the *n_H_* range expected for 1:1 binding in the shaded area. (**D**) The case of an apparent 1:1 binding simulated for 1000 molecules (light purple) for σ = 2 kJ mol^−1^, i.e. minimal binding heterogeneity. The mean binding isotherm is shown in circles together with a 1:1 fit (black curve). (**E**) Same as panel D but for σ = 4.5 kJ mol^−1^ corresponding to a scenario with substantial binding heterogeneity. The thin black curve is the shape of the isotherm expected for 1:1 binding without any heterogeneity in binding free energies. (**F**) Distribution of binding free energies in an ensemble of 1000 molecules derived from the heterogeneity analysis (see main text) for the temperatures shown in panel A.

The full-length CytR (that includes the DNA-binding and oligomerization domain) is dimeric and highly promiscuous with the ability to bind octamer *udp* half-sites with multiple inter-repeat spacing and orientations (38,39). This promiscuity has been attributed to the disordered nature of CytR DNA-binding domain (40). Does the change in the dimensions of CytR DNA-binding domain affect DNA-binding ability? Binding studies with fluorescently labeled *udp* half-site reveal broad binding isotherms that do not saturate in the temperature range of 278–308 K with the anisotropy values continually increasing with CytR concentration (circles in Figure 5A). Accordingly, a 1:1 equilibrium model does not provide a statistically convincing fit to the data (Supplementary Figure S6; a poor 1:1 fit is also observed in the original CytR article (40)).

We therefore resorted to the Hill binding equation (52) to extract the *K*_1/2_ values (midpoint of binding) and the Hill coefficients (*n_H_*) that report on binding cooperativity:

\begin{equation*}f = \frac{{{{[CytR]}^{{n_H}}}}}{{K_{1/2}^{{n_H}} + {{[CytR]}^{{n_H}}}}}\end{equation*}

where *f* is the fraction of ligand bound. It fits the binding isotherms very well (lines in Figure 5A) with the midpoint of binding shifting to the left with increasing temperatures (∼23 μM at 278 K to ∼9 μM at 308 K, Figure 5B), which is unusual given that either parabolic trends or weakening affinities are generally observed with temperature. The Hill coefficients are surprisingly less than 1 in this temperature range, 0.60 ± 0.02 at 278 K to 0.69 ± 0.02 at 308 K, and marginally increase with temperature (Figure 5C). A possible explanation for such ‘negative cooperativity’ is when two or more CytR monomers bind the *udp* half-site. However, NMR experiments, through which the structural model of CytR was developed, clearly indicate that binding is monomeric even up till a CytR concentration of 400 μM (40); this is four times higher than the maximal concentration employed in our experiments (∼100 μM; Figure 5A), thus ruling out higher order effects. Cooperativity effects from ensemble measurements and in single-ligand binding are rare (though frequently reported in enzyme kinetics (53)) and, to our knowledge, this is first such instance reported for protein-DNA binding.

What feature determines the apparent negative cooperativity in a 1:1 binding? It is important to note that the Hill equation and the associated mechanistic interpretation of the stoichiometry is valid only when the ligand is fully folded or when the binding pose is invariant or well-defined (54). Physically, Hill coefficients are the second moments of binding energy distributions (54,55) and therefore in 1:1 binding scenarios *n_H_* < 1 represents binding heterogeneity. Since CytR is disordered under these conditions, the apparent negative cooperativity reported here should be a consequence of the associated heterogeneity with multiple conformations or orientations of the folded domain binding DNA, with the NMR structure corresponding to dominant binding mode. In this regard, it is interesting to note that single molecule experiments on ion-dependent folding of P4–P6 RNA reveal that though the bulk Hill coefficient is close to 1, the parent single-molecule binding isotherms exhibit a large spread in folding-binding free energies (56). The corollary is that bulk binding isotherm can be thought of as a superimposition of multiple isotherms from single molecules binding with varying affinities (i.e. binding heterogeneity), the effective sum of which translates to a slow rise in experimental anisotropy with the ligand concentration.

To extract the extent of binding heterogeneity that manifests as *n_H_* < 1, we first assume that the experimental binding free energy of CytR to DNA from individual molecules is normally distributed (and as observed in single-molecule experiments (56,57)). We then modulate the standard deviation (σ) of this distribution, simulate individual 1:1 binding isotherms picking binding free energies values from the normal distribution, average them to extract an apparent bulk binding isotherm and identify the width that best reproduces the experimentally observed Hill coefficients and *K*_1/2_ values. *n_H_* values distinguishable from 1 (<0.9 as experimental errors also contribute to the variability) are observed only when σ is >2–2.2 kJ mol^−1^ (Figure 5D) and are challenging to identify as they are comparable to the thermal energy. In the case of CytR, the experimental Hill coefficients can be recovered (Supplementary Figure S7) when the width of the underlying binding energy distributions are substantially larger than thermal energy, ranging from ∼5.1 kJ mol^−1^ at 278 K to ∼4.5 kJ mol^−1^ at 308 K (Figure 5E and F).

## DISCUSSION

Our results reveal that the observed complex binding isotherms arise from intrinsic heterogeneity in binding that in turn can be attributed to the conformational landscape of CytR. What is the functional advantage? It is well known that a dynamic equilibrium between non-specific and specific binding modes, for ‘search’ and ‘recognition’, respectively, needs to exist in DNA-binding proteins to enable efficient search of target sites (58–61). Simulations reveal that 1D sliding motions on DNA, that dramatically increase the identification of target sites (i.e. facilitated diffusion (58)), tune the affinity/specificity of DNA binding (62) and increase with increasing disorder and degree of one-state-like character (‘downhillness’) in the underlying conformational ensemble (63). This intricate requirement is exacerbated in CytR, as it not only collapses with temperature but also folds upon binding DNA.

It is therefore tempting to speculate that the collapse transition is functionally driven to enable access to binding conformational pose(s) (akin to the ‘conformational selection’ mechanism) through large thermodynamic fluctuations within the flat downhill-like free energy landscape at near the optimal bacterial growth temperature. This is particularly evident in the plots of *R_g_* versus *Q* derived from implicit solvent simulations wherein CytR is shown to sample a large array of conformations some of which are folded-like at 310 K (high *Q* and low *R_g_* in sub-ensemble *b* in Figure 4J). Such a feature will proportionately minimize non-specific binding and is consistent with our observations of a correlation between an increase in binding affinity and (marginal) decrease in binding heterogeneity with temperature (Figure 5B and C). A similar observation has also been made on Zinc-finger binding domains with an increased binding affinity correlated with a population shift towards the ‘recognition’ mode (64).

Temperature-induced coil to globule collapse transitions in unfolded or intrinsically disordered proteins have generally been studied for understanding the balance of energetic terms determining folding, to optimize force-fields and to explore the limits and applicability of various scaling laws in polymer physics. Given that simulations and experiments on disordered states almost always point to collapse with temperature or changing solvent conditions, it is natural to expect this feature has evolved for functional purposes. In this work, we show that the phase space accessible to CytR is highly heterogeneous and dependent on the immediate solvent conditions: at low temperatures, the protein is expanded and exhibits random-coil statistics while at high temperatures it collapses to a globule exhibiting large thermodynamic fluctuations but stabilized by non-native hydrogen bonds. The CytR conformational ensemble at the optimal bacterial growth temperature is precisely balanced between both compact and coil-like conformations (i.e. the Θ-state) thus exhibiting ideal-chain dimensions and maximal enthalpic fluctuations for potential functional reasons. The observed negative binding cooperativity to single-site DNA is a direct consequence of the diversity in molecular dimensions translating into varying binding affinities, similar to that reported in the disordered region of the Notch receptor protein (65).

It is also possible that CytR folds and binds with multiple orientations, each pose exhibiting a different binding affinity to the *udp* half-site. In fact, significant and diffuse electrostatic frustration is observable in the DNA-binding face of CytR (from the structure of the DNA-bound state, Supplementary Figure S8); this feature potentially highlights why CytR is disordered in the absence of DNA, the role of DNA in determining the conformational landscape of CytR and the possible reason for heterogeneous binding through multiple electrostatically equivalent orientations. Structurally, this hints that an array of binding modes is accessible to CytR, some which are specific and some non-specific, a characteristic feature of even structured DNA-binding proteins (60,64,66,67), that is visibly exaggerated due to a combination or conflicts from collapse, large enthalpic fluctuations and DNA-driven folding. The non-specific poses should enable CytR to bind even random DNA sequences within the bacterial genome with the degree of structural ordering driven purely by the extent of similarity of the numerous DNA sequences with the specific site. In other words, the conformational space sampled by CytR could be determined by the DNA sequence-structure to enable a precise balance between one- and three-dimensional diffusive modes facilitating an effective search of target sites.

## Supplementary Material

Supplementary Data
