# Supplementary material for: Protein plasticity driven by disorder and collapse governs the heterogeneous binding of CytR to DNA
Source: Nucleic Acids Res. 2018 Mar 10;46(8):4044–53. doi: 10.1093/nar/gky176 (PMC5934615; doi:10.1093/nar/gky176)
Supplement: Supplementary Data [file gky176_supp.pdf]

# Supplementary Data

# Protein Plasticity driven by Disorder and Collapse governs the Heterogeneous Binding of CytR to DNA

*Sneha Munshi,[1] Soundhararajan Gopi,[1] Sandhyaa Subramanian,[1] Luis A. Campos[2] & Athi N. Naganathan[1]**

[1]Department of Biotechnology, Bhupat & Jyoti Mehta School of Biosciences, Indian Institute of Technology Madras, Chennai 600036, India.

[2]National Biotechnology Center, Consejo Superior de Investigaciones Científicas, Darwin 3, Campus de Cantoblanco, 28049 Madrid, Spain.

e-mail: athi@iitm.ac.in

## Supplementary Methods

***Protein Expression and Purification*** The gene for CytR corresponding to the protein sequence MKAKKQETAATMKDVALKAKVSTATVSRALMNPDKVSQATRNR VEKAAREVGYLPQPMGRNVKRNE was cloned in to the pTXB1 vector containing chitin binding domain (IMPACT$^{TM}$ from New England Biolabs) and transformed into the BL21 (DE3) expression system. The cells were grown at 37°C, induced with 2 mM IPTG at an OD of ~1.2 and further grown for 2 hours. The cells were then harvested, lysed, centrifuged and the supernatant was passed through 5 ml of chitin resin column and washed with 200 mM sodium phosphate buffer (pH 8.0) to remove impurities. The cleavage reaction was initiated by equilibrating the column in the wash buffer with 50 mM β-mercaptoethanol and incubated for 12-14 hours for the reaction to complete. The cleaved protein was eluted from the column in 50 mM phosphate buffer (pH 8.0), passed through a cation exchange column and finally purified through a HiLoad 16/600 Superdex 75pg column (GE Healthcare, USA equilibrated with 150 mM ammonium acetate buffer, pH 8.0) employing a AKTA Start preparative chromatography system. The fractions containing purified CytR were pooled and lyophilized. PurR (sequence: MATIKDVAKRANVSTTTVSHVINKTRFVAEETRNAVWAAIKELHYSPSAVAR SLKVN) purification protocol is similar to the methodology above except that *E. coli* ER2566 cells were used as the expression system, 20 mM sodium phosphate, 50 mM NaCl (pH 8.5) for the chitin column wash/elution buffer and a HiLoad 26/600 Superdex 30pg column (GE Healthcare, USA) was employed in the final purification step.

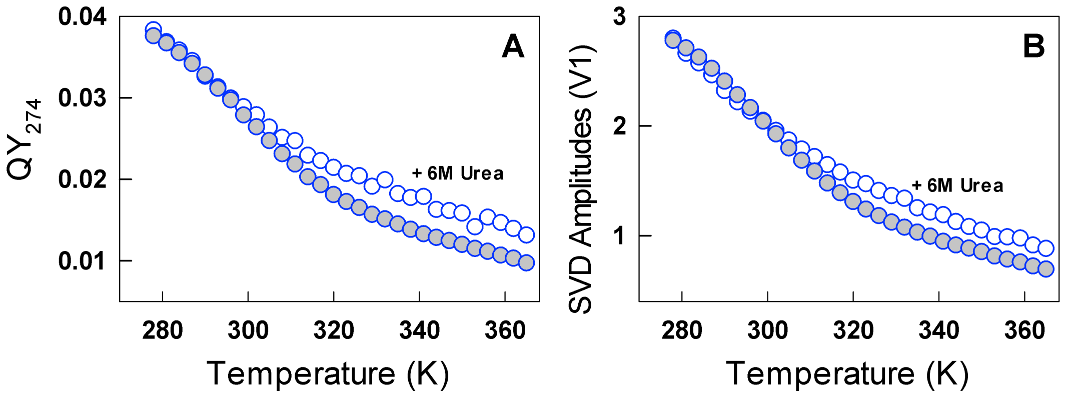


**Figure S1.** (A) The quantum yield (QY) of CytR in the presence (filled circles) and absence (open circles) of 6 M urea. (B) The temperature dependence of the first component (black in Figure 2D) of a global SVD of the temperature-wavelength data as discussed in the main text.

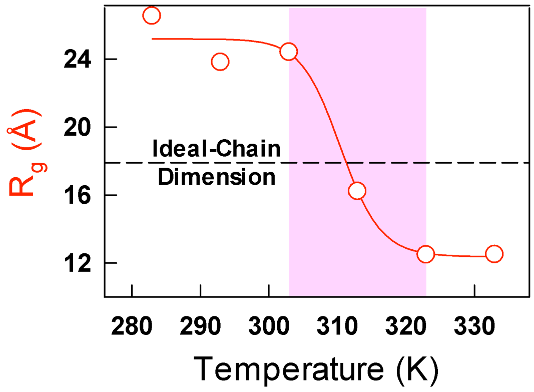


**Figure S2.** Changes in chain dimensions as reported by radius of gyration ($R_g$) extracted from the hydrodynamic radius (Figure 1B of the main text). The ideal-chain dimension (corresponding to the Θ-point) is 17.9 Å and is shown as a horizontal dashed line. The shaded area represents the collapse transition regime.

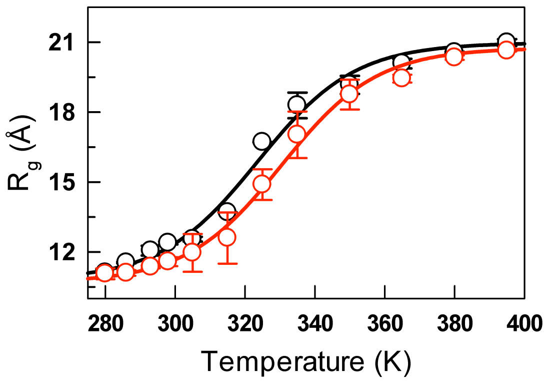


**Figure S3** Changes in dimensions of CytR (radius of gyration, $R_g$) as a function of temperature with temperature-dependent (red) and temperature-independent dielectric constants (black) using the ABSINTH implicit-solvation model. Temperature-dependent solvation free energy was not introduced in either of the simulation set-up.

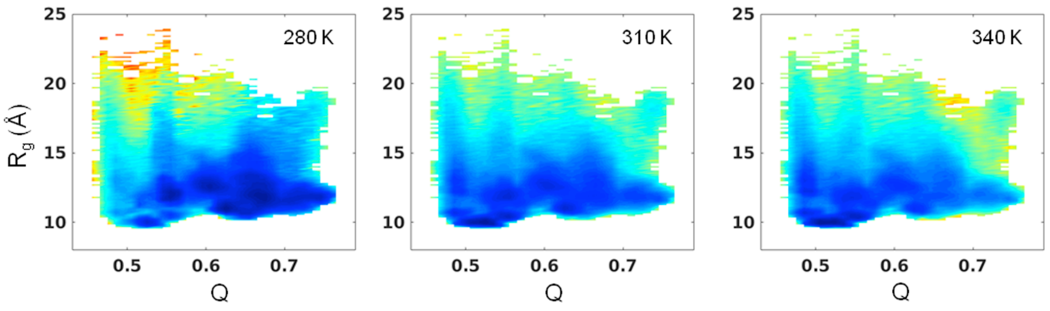


**Figure S4** Two-dimensional free energy surfaces of CytR at different temperatures spanning the collapse transition derived from the all-atom replica-exchange Monte Carlo simulations. A spectral color-coding is employed that goes form blue (low in free energy) to red (high). The order parameters are the radius of gyration ($R_g$) and fraction of native contacts ($Q$; with respect to the folded structure). Note that the highly populated clusters (dark blue) collapse and unfold (lower $Q$) more with increasing temperatures.

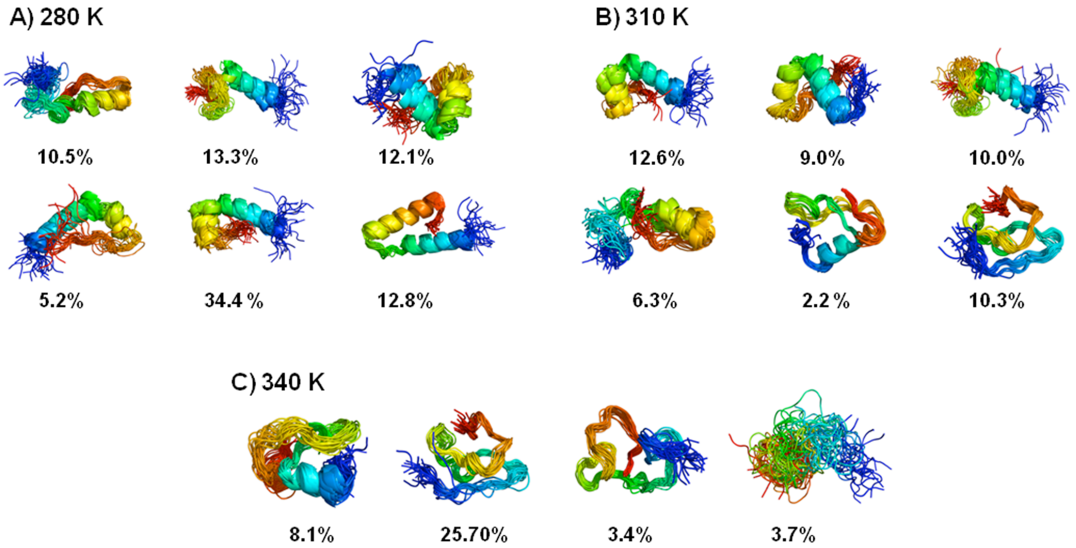


**Figure S5** Snapshots of the sub-ensembles (with 28-31 frames in each cartoon) at different temperatures and their populations. Compact but unstructured globules can be seen at 340 K contrasting with the ensemble at 280 K. At 310 K, both the extreme structural sub-ensembles are evident.

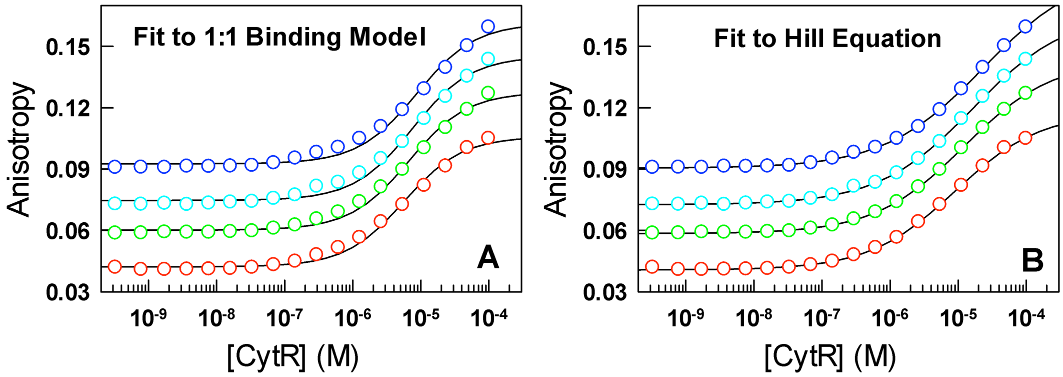


**Figure S6** Comparison of the 1:1 (panel A; black curves) and Hill-equation fits (panel B; black curves) to the experimental binding isotherms (circles) at different temperatures. It can be clearly seen that the Hill equation fits visually better. The *F*-statistic is more than 200 in all cases, corresponding to a $p$-value $< 10^{-14}$. F-test therefore suggests that the more complex Hill equation also fits the experimental data statistically better than a simple 1:1 binding model. The temperatures are 278 K (blue), 288 K (cyan), 298 K (green) and 308 K (red).

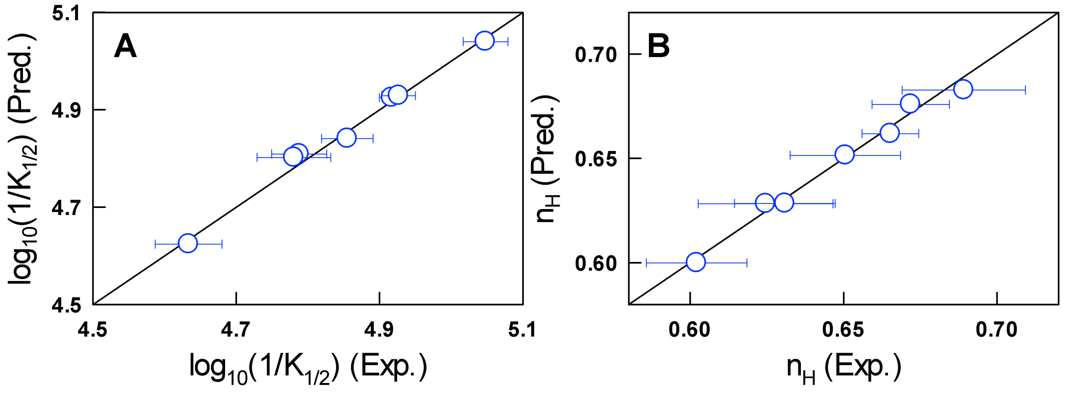


**Figure S7** Experimentally derived $K_{1/2}$ and $n_H$ values (Exp.) compared against that derived from the simulated average binding isotherm from 1000 molecules (Pred.; with 1:1 binding affinity that is normally distributed; Figure 5 in the main text). The perfect correlation line is shown in black. The fitting errors for the simulated binding isotherms are smaller than the circle dimensions.

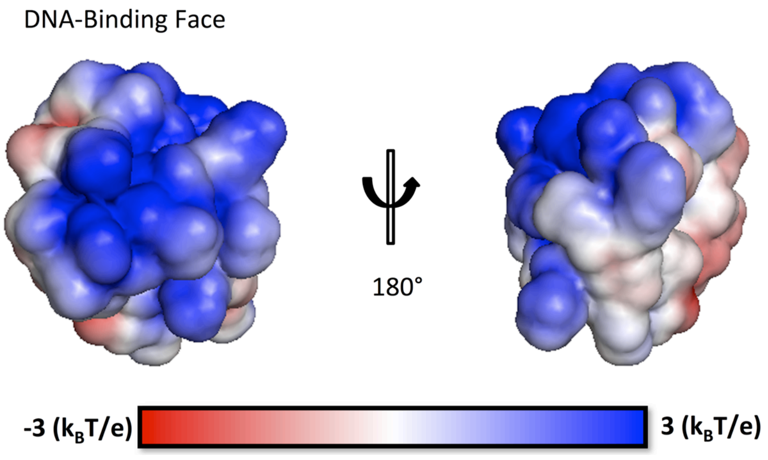


**Figure S8** Electrostatic frustration in the DNA-binding face of folded CytR.
